# Postoperative leg length discrepancy after hip and knee arthroplasty induces measurable TMJ changes without clinical dysfunction

**DOI:** 10.1186/s12891-026-09772-3

**Published:** 2026-04-14

**Authors:** Fabian Fenske, Jakob Schmidt, Anna Katharina Sander, Andreas Maximilian Fichter, Oliver Schierz, Andreas Roth, Christina Pempe, Elisabeth Grau

**Affiliations:** 1 Department of Oral and Maxillofacial Surgery, University Hospital Leipzig, Liebigstr. 12, Leipzig, 04103 Germany; 2 Department of Prosthetic Dentistry and Materials Science, University of Rostock, Rostock, Germany; 3 Department of Orthopedics, Traumatology and Plastic Surgery, University Hospital Leipzig, Leipzig, Germany; 4 Department of Oral and Maxillofacial Surgery, Charité – Universitätsmedizin Berlin, Corporate Member of Freie Universität Berlin and Humboldt-Universität zu Berlin, Augustenburger Platz 1, Berlin, 13353 Germany

**Keywords:** TMD, TMS, Temporomandibular system, Leg length compensation, TKR, THR, Leg length discrepancy, LLD

## Abstract

**Background:**

This study investigates the influence of postoperative leg length discrepancy (LLD) on the temporomandibular system (TMS) after total hip or knee replacement (THR/TKR), focusing on long-term effects and functional or positional changes in the temporomandibular joint (TMJ).

**Methods:**

A total of 45 patients (31 THR, 14 TKR) from Leipzig University Hospital were prospectively examined preoperatively and after 8–12 weeks. LLD was measured and categorized. A control group of 20 subjects without surgical interventions was used to compare the initial situation, and oral health was assessed using OHIP-G14 questionnaires. Statistical analysis was performed using SPSS version 24. Level of significance was accepted at *p* < 0.05.

**Results:**

While no patient developed temporomandibular disorders (TMD), functional and positional changes in the TMS were observed postoperatively, particularly in patients with a LLD ≥ 15 mm. Significant changes included increased mouth opening (+ 4.3 mm) and decreased left laterotrusion (-1.98 mm) in the high compensation group. Shifts in condylar position were noted in all spatial axes. No significant changes in function were observed in the control group and in patients with a LLD ≤ 10 mm. The subjective oral health-related quality of life remained unchanged.

**Conclusions:**

Postoperative LLD after THR or TKR leads to measurable changes in TMJ position and mandibular function. However, these changes did not lead to clinical TMD within the study period.

**Trial registration:**

This study involved examinations only and did not include any intervention. Therefore, trial registration was not required and was not performed. Clinical trial number: not applicable.

## Introduction

The human body maintains health through complex physiological interactions and balance between its systems. This principle also applies to the temporomandibular system (TMS) and its relationship to the rest of the body. Clinical complaints in this system, known as temporomandibular disorders (TMD) affect about 5–31% of the population and represent the main cause of non-dental orofacial pain, often accompanied by symptoms such as earache, cephalgia, neuralgia or toothaches [1–4]. The bidirectional influence of the TMS and the body has a multifactorial pathogenesis and remains a subject of ongoing debate [5].

TMD clinically presents with joint noises, restricted mandibular movement, and muscle pain on palpation. Further studies reported links to conditions in more distal regions of the body, such as chronic back pain or gastrointestinal diseases [6]. Accordingly, modern concepts move beyond the gnathological paradigm that focused primarily on occlusion and the temporomandibular complex [7]. The search for potential etiological factors of TMD therefore also extend to the specialties of neurology, rheumatology, internal medicine, psychology, and especially orthopedics [8, 9].

In this context, total hip replacement (THR) and total knee replacement (TKR) are of special interest. These procedures are performed with high frequency in the countries of the Organization for Economic Co-operation and Development (OECD): in 2011, approximately 400,000 cases were documented in Germany, 1.4 million in the United States, 200,000 in the United Kingdom, and 230,000 in France [10, 11]. More recent data suggests a total of 233,000 THRs and 187,000 TKRs in Germany in 2016 alone [12].

Postoperative leg length discrepancies (LLD) after hip and knee implants occur as a common complication, reported in up to 80% of patients [13, 14]. LLD refers to a structural or functional difference in the length of the lower extremities, which can lead to altered biomechanical stress and possible musculoskeletal complaints. It may be anatomical, functional, or subjectively perceived by the patient – the latter being frequently reported even when not confirmed by clinical diagnostics or imaging modalities. Importantly, perceived LLD correlates negatively with patients’ functional outcomes [15].

Such LLD has a variety of clinical consequences, such as chronic back pain, gait disturbances, painful asymmetries of muscle chains, or sciatica syndrome, all of which can be attributed to altered body posture [16–19]. Whether such postural changes exert downstream effects on remote regions such as the TMS, in line with the principles of ascending and descending musculoskeletal chains, remains controversial. While interest in these correlations is high, evidence from high-quality studies is limited [9, 20, 21].

Previous studies primarily examined experimentally induced LLD in terms of the board method or recorded only short-term changes within a few days after surgery [22–24]. However, the studies do not depict long-term adaptions of the TMS or capture the clinical relevance of these changes. Moreover, studies have frequently restricted outcome measures to the condylar position or mandibular movements, overlooking broader functional changes and systemic interactions [25]. In summary, relevant preliminary studies show that experimentally or postoperatively induced leg length differences and changes in posture are associated with measurable changes in jaw position, mandibular movements, and upper body statics. However, these have been studied predominantly in the short term and have not yet been able to demonstrate clear clinical relevance in terms of manifest TMD.

Therefore, the aim of this study was to investigate whether postoperative LLD after THR and TKR leads to measurable changes in TMJ position and mandibular function, especially in longer terms. In addition, we sought to determine whether these changes have clinical relevance in terms of TMD development, to what extent they should be treated, and whether they may inform therapeutic strategies and interdisciplinary patient management.

## Materials and methods

This prospective study was conducted at the Leipzig University Hospital over a 10-month period. All patients provided written informed consent prior to the study participation. The study complied with the standards of the Declaration of Helsinki and was approved by the Ethics Committee of the University of Leipzig (No. 403/21-ek).

### Study participants

A total of 45 patients undergoing total hip replacement (THR) or total knee replacement (TKR) were enrolled. Inclusion criteria were age ≥ 18 years and sufficient dentition, defined as Eichner classification B2 or higher, ensuring at least two supporting zones. Exclusion criteria were infectious diseases and pregnancy.

Additionally a control group of 20 subjects was examined prior to this study to validate the measurement method and allow for later comparison [26]. None of the controls underwent surgery and consequently showed no change in leg length.

Data collection was held at three different points in time and was performed by two alternating investigators. The patients were examined preoperatively, three to five days postoperatively (after sufficient inpatient mobilization), and after rehabilitation within a period of eight to twelve weeks. In each of the three appointments, changes in leg lengths as well as digital instrumental analysis of condylar position and mandibular movements were recorded.

### Questionnaires and clinical examination

Oral health-related quality of life was assessed using the OHIP-G14 questionnaire [27]. General health-related quality of life was recorded using the Short Form-36 (SF-36) [28]. In addition, baseline diagnostics for temporomandibular disorders (TMD) were performed according to the guidelines of the German Society for Functional Diagnostics and Therapy (DGFDT) [29]. Where indicated, the DC/TMD questionnaire was administered. This was required if several of the screening criteria were positive or if one of the severe signs was present.

### Assessment of leg length discrepancy and the temporomandibular system (TMS)

Leg length discrepancy (LLD) was measured pre- and postoperatively as the distance between the anterior superior iliac spine and the lateral malleolus bilaterally. In addition, direct assessment was performed using a pelvic leveling device and calibrated wooden blocks placed under the shorter leg until pelvic balance was achieved.

Mandibular movements and condylar positions were recorded using an optoelectronic registration system (Tizian JMA Optic, Zebris Medical GmbH, Isny, Germany). The system can mathematically determine the terminal hinge axis and thus the relevant information for the function. The condylar position was determined using the “Electronic Position Analysis” (EPA) measurement mode of the JMA Optic. In maximum intercuspation, this position can be considered unchanged, since no occlusal intervention was performed between the measurements. As cranial reference point, served the tragus, entered into the system using a pointer. For the measurement of the reference position the mandibular hinge axis was determined in maximal occlusion. This position served as a reference position. The hinge axis was determined as the functional center of rotation of the condyle using minor opening movements. This was compared pre- and post-surgically to the rest position of the temporomandibular joints.

**Fig. 1:**
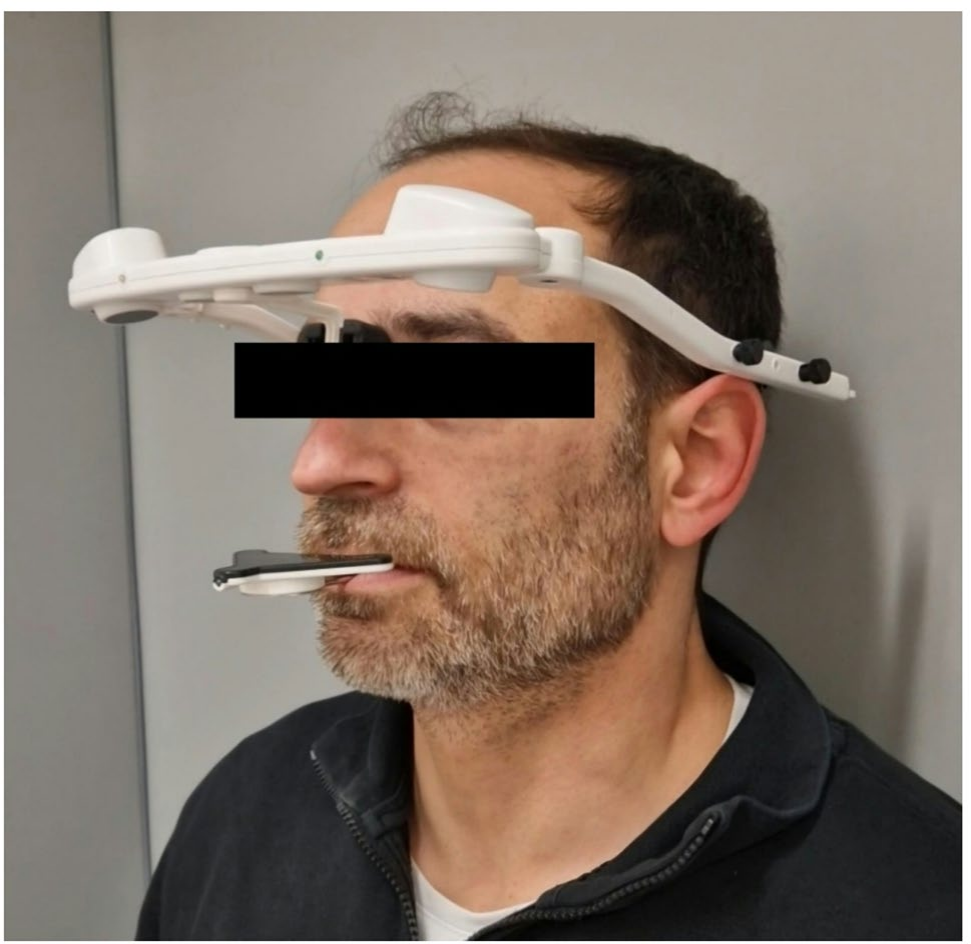
Test subject with measuring instrument attached

Minimal opening movements do not result in translation, but rather in rotation, which is assumed here to be pure rotation. This allows the system to determine the hinge axis around which the mandible rotates and thus determine the functional center of rotation of the condyle. This procedure has been validated by a study using the same device [26]. In summary, the JMA-Optic does not determine the condyle position by imaging; rather, it calculates it as the kinematically optimal axis of rotation from the 3D movement of the lower jaw relative to the skull during very small opening movements. The system operates on the principle that an infrared signal controls a sensor, that is magnetically attached to paraocclusal mounts in the mandible. These attachments were temporarily cemented to the mandibular teeth using a bis-acrylate (DMG Dental, LuxaTemp). The cameras integrated in the facebow recorded the movements or positions and recalculated them to the entire mandible. Figure 1 shows the setup of an examination of the TMS. Measurements included maximum mouth opening, right and left laterotrusion, and protrusion. Condylar positions were assessed in all three spatial planes after calibration in the physiologic mandibular rest position. Each measurement was repeated five times, and mean values were calculated. Data were stored and analyzed using the JMT Function Pro software (v1.4.19 Schütz Dental GmbH, Rosbach, Germany).

The primary outcomes were mandibular movements and condylar position changes following THR or TKR. Secondary outcomes included oral health impact profile (OHIP-G14), general health-related quality of life (SF-36), and clinical findings of TMD. The OHIP-G is a validated questionnaire for assessing oral health-related quality of life, which measures the subjective impairment caused by oral complaints in various dimensions. If the score remains unchanged before and after surgery, this indicates that the procedure had no measurable impact on the patient’s perceived quality of life.

### Statistical analysis

Statistical analyses were performed using SPSS for Windows (Version 24.0, IBM Corp., Armonk, NY, USA.). Metric variables were presented as means and medians, whereas dispersion measures were presented as standard deviations (SD) and quartiles. Metric variables were tested for normal distribution using the Kolmogorov-Smirnov test. The tested variables did not show normal distribution (*p* < 0.05). Consequently, non-parametric tests were used throughout the sample comparisons.

When comparing two independent samples, the Mann-Whitney U test was applied. When comparing more than two independent samples, the Kruskal–Wallis H test was performed. The categorized or nominal data, on the other hand, were analyzed using the chi-square test. All tests were two-sided, and statistical significance was set at *p* < 0.05. Boxplots and bar charts were generated to visualize the results.

## Results

### Patient characteristics

The cohort comprised 45 patients of which 14 received a TKR (8 right, 5 left, 1 bilateral) and 31 were inserted a THR (19 right, 12 left).

The mean age of patients was 65.27 years (SD 8.87) and the cohort comprised 26 women (57.8%) and 19 men (42.2%), of whom four were left-handed (8.9%) and four were left-footed (8.9%).

The control group consisted of 12 men and 9 women, with 20 right-handers and 1 left-hander within this group. There were 16 right-footed and 5 left-footed people and the average age was 42.9 years. One control subject was excluded from three-dimensional condylar analysis due to the inability to achieve a reproducible occlusal position.

### Leg length discrepancy

The compensation of the LLD was ≤ 10 mm in 36 patients and ≥ 15 mm in 9 patients. The mean compensation was 7.67 mm (median 5 mm, range 0–40 mm). None of the patients presented clinical signs of TMD before or after surgery. Compensation refers to the change in leg length discrepancy (LLD) quantified as the difference between preoperative and postoperative measurements, reflecting the amount of surgical correction achieved. Table 1 presents the distributions and mean compensation according to the preoperative LLD.

**Table 1:** Distribution and mean compensation stratified by preoperative leg length discrepancy (LLD)

LLD preoperative (mm)	0	1–5	6–10	> 11	Total
% (*N*)	16 (7)	24 (11)	42 (19)	18 (8)	100 (45)
Female (N)	3	7	10	6	26
Right handed (N)	5	10	18	8	41
Right footed (N)	6	11	17	7	41
Mean compensation (mm) (*SD*)	3.57 (3.78)	4.09 (3.02)	7.63 (7.14)	16.25 (11.57)	7.67 (8.09)

### Oral health and quality of life

No significant differences were found when patients were asked about their oral health pre- and postoperatively using the Oral Health Impact Profile (OHIP-G). Low values were determined at both points in time with a mean of 0.67 preoperatively and a mean of 1.14 eight to twelve weeks postoperatively. In addition, the screening for TMD did not reveal any abnormalities in any of the patients, and therefore no further diagnostics were indicated.

### Mandibular movements

We decided to compare the results of the first appointment with those of the third examination. The values obtained at the second examination were excluded due to inadequate quality since patients were not yet sufficiently mobilized.

When measuring the movements of the mandible with the JMA Optic in the function measurement mode, no significant differences were found when comparing the control group with the changes in the 0–10 mm compensated group. In the group of patients who were compensated 15–45 mm, there was a significant increase in mouth opening by an average of 4.3 mm (95% CI: 0.90–7.70; *p* < 0.05). Laterotrusion to the left changed significantly with a mean decrease of 1.98 mm (95% CI: -3.28–0.68; *p* < 0.05). Table 2 presents the functional movements of the mandible depending on the compensated LLD.

**Table 2:** Change scores in functional mandibular movements stratified by compensated LLD

Group/ LLD compensation	Control (mm) (SD)	0–10 mm (mm) (SD)	15–40 mm (mm) (SD)
Opening	0.30 (2.43)	0.23 (4.23)	4.30 (4.43)*
Laterotrusion right	0.11 (1.35)	0.18 (1.79)	0.77 (1.33)
Laterotrusion left	0.26 (1.16)	0.90 (1.96)	-1.98 (1.69)*
Protrusion	-0.14 (2.09)	-0.15 (3.96)	0.30 (2.57)

*p  < 0.05

### Condylar position

The condylar position was determined using the “Electronic Position Analysis” (EPA) measurement mode of the JMA Optic. Small but significant shifts were observed between the position of the hinge axis in patients with LLD 0–10 mm in all three axes bilaterally (range 0.34–0.78 mm, *p* < 0.05). In the right TMJ, the position changed by 0.34 mm (95% CI: 0.19–0.49; *p* < 0.05) on the X-axis, 0.78 mm (95% CI: 0.51–1.05; *p* < 0.05) on the Y-axis and 0.63 mm (95% CI: 0.40–0.87; *p* < 0.05) on the Z-axis. In the left joint, the position changed by 0.33 mm (95% CI: 0.18–0.48; *p* < 0.05) on the X-axis, by 0.56 (95% CI: 0.35–0.78; *p* < 0.05) mm on the Y-axis and by 0.47 mm (95% CI: 0.28–0.66; *p* < 0.05) on the Z-axis.

In patients with a LLD of 15–45 mm, larger positional changes occurred. The position of the right TMJ changed with a mean of 1.24 mm (95% CI: 0.71–1.76; *p* < 0.05) on the Y-axis and 0.69 mm (95% CI: 0.14–1.25; *p* < 0.05) on the Z-axis. The change on the left side was significant in the same axes, namely on the Y-axis by 1.05 mm (95% CI: 0.15–1.95; *p* < 0.05) and on the Z-axis by 0.67 mm (95% CI: 0.26–1.09; *p* < 0.05).

Considering the operated side, in relation to the 0–10 mm and the 15–45 mm group, the evaluated changes ipsi- and contralaterally showed significant results only in one axis. This was 0.7 mm (95% CI: 0.49–2.25) ipsilaterally on the Y-axis (*p* < 0.05). Table 3 shows how the three-dimensional position of the condyle has changed in relation to the compensated LLD.

**Table 3:** Position of the joint stratified by compensated LLD

Group/ LLD Compensation	Control Mean (mm) (SD)	0–10 mm (mm) (SD)	15–40 mm (mm) (SD)
x-axis (left joint)	0.08 (0.09)	0.33 (0.44)*	0.78 (1.23)
x-axis (right joint)	0.08 (0.07)	0.34 (0.45)*	0.50 (1.04)
y-axis (left joint)	0.14 (0.14)	0.56 (0.64)*	1.05 (1.17)*
y-axis (right joint)	0.18 (0.19)	0.78 (0.80)*	1.24 (0.68)*
z-axis (left joint)	0.17 (0.09)	0.47 (0.56)*	0.67 (0.54)*
z-axis (right joint)	0.18 (0.31)	0.63 (0.69)*	0.69 (0.72)*

* *p* < 0.05

## Discussion

This prospective study demonstrated that postoperative leg length discrepancies (LLD) after total hip or knee arthroplasty is associated with measurable changes in temporomandibular joint (TMJ) position and mandibular function across all spatial axes, particularly when the discrepancy was ≥ 15 mm. While no clinically relevant changes were observed in the control group and in cases of minor LLD (≤ 10 mm), more pronounced LLD was associated with a significant increase in mouth opening and reduced lateral movement to the left. These findings support the concept of ascending musculoskeletal adaption and indicate that the stomatognathic system adapts to orthopedic changes in the lower extremities. Importantly, no patient developed a clinically manifest temporomandibular disorder (TMD) during the 8–12-week follow-up.

The demographic characteristics of our cohort (mean age 65 years; 58% female) are consistent with typical arthroplasty populations and comparable to previous reports [30]. In our cohort, 20% of patients were found to have a postoperative LLD of ≥ 15 mm, with an average compensation of 7.67 mm. This is consistent with previous studies reporting that LLD is common after THR and TKR. In particular, a systematic review found that the incidence of radiographically detectable LLD after TKR ranged from 44 to 83%, with an average leg lengthening of 5.98 mm [31]. After THR, LLD ≥ 10 mm was found in 15–16% of patients [32]. These deviations could be due to differences in surgical methods, preoperative planning, and intraoperative techniques, and also include implant selection [31].

The change in position of the condyle found in our study can be reconstructed with the adaptions already described in existing literature, as the adaption via the pelvis, spine and the corresponding cervical spine has been described previously [23, 25, 33–35]. Not only a change in the position of the temporomandibular joint, but also a change in function has been demonstrated, whereby an increased maximum mouth opening has already been linked to orthopedic changes in one study [3]. Improved posture led to an increase in mouth opening, which may also have occurred after surgery in our subjects, including compensation for LLD [3].

The LLD that develops after surgery can therefore have far-reaching consequences, which have also been described in other studies, whereby an LLD of 10 mm or more often plays a decisive role. This threshold value has proven useful in the literature, as it is associated with functional and biomechanical impairments after surgery as well as pain in the lower back [3, 13, 16, 17]. Iwakiri et al. showed that achieving a LLD of less than 1 cm significantly improved the patient’s perception [17]. For this reason, the groups in this study were divided into those with less than and more than 10 mm LLD. The fact that the group in which greater orthopedic changes were made also experienced the most changes in TMS is thus consistent with the existing evidence. One study by Komiyama et al. examined patients with TMD who underwent orthopedic training and confirmed the association [36]. In the trained group, a change in posture contributed to a significant improvement in mouth opening [36].

The principle of the ascending chain can therefore be supported, however none of our study subjects developed TMD. This absence of clinically diagnosed CMD cases must be interpreted with caution. The study cohort was relatively small, and the follow-up period may have been too short to capture later dysfunction. Thus, while postoperative LLD induced subclinical changes in TMJ function and position, our data cannot exclude the possibility of subsequent clinical manifestations. Since no significant changes can be detected in the OHIP-G evaluation, this supports the study’s conclusion that no clinical relevance can be inferred. The adaption has already been investigated in the literature, but the measurements were taken a few days postoperatively without sufficient mobilization and adaption in the ascending chain [22, 24]. It is therefore the strength of this study that the postoperative examination took place at a later point in time. One limitation of the study is that the observation period may not be long enough to detect late-onset TMD. Further investigation in the topic and a larger sample size will be required to obtain more evidence.

Authors describe not only an ascending but also a descending chain with influence of TMS on orthopedic areas [8, 20, 37, 38]. Manfredini et al. point out that the interrelationship between occlusion, posture, and TMD is complex but should not be neglected, and emphasize the importance of a biopsychosocial model [8]. Armijo Olivo et al. also emphasize the close functional connection between the cervical spine, stomatognathic system, and craniofacial pain in their critical review [20]. This interaction should be taken into account by physicians, and interdisciplinary cooperation is therefore to be advocated, as it has already been recognized in other studies [34, 39, 40].

Taken together, our findings confirm the kinematic link between lower extremity alignment and TMJ position and function. They underline the need for interdisciplinary awareness between orthopedic and dental specialties, particularly in patients with pronounced postoperative LLD.

## Conclusion

Postoperative LLD after THR and TKR leads to measurable changes in rest position of the temporomandibular joint and mandibular function, particularly when the discrepancy exceeds 15 mm. These alterations reflect ascending musculoskeletal adaption but did not result in clinically manifest TMD within a follow-up period of 8–12 weeks. Larger longitudinal studies are needed to clarify the long-term clinical relevance and to guide interdisciplinary management between orthopedics and dentistry.

## Data Availability

The datasets used and analysed during the current study are available from the corresponding author on reasonable request.

## Abbreviations

DGFTD: German society for functional diagnostics and therapy
LLD: Leg length discrepancy
OECD: Organization for economic co–operation and development
OHIP: G–Oral health impact profile
SD: Standard deviation
THR: Total hip replacement
TKR: Total knee replacement
TMD: Temporomandibular disorders
TMS: Temporomandibular system
SF-36: Short form–36
